# Cytomegalovirus-related uncontrolled glaucoma in an immunocompetent patient: a case report and systematic review

**DOI:** 10.1186/s12886-018-0917-9

**Published:** 2018-09-29

**Authors:** Lei Xi, Liang Zhang, Wenlei Fei

**Affiliations:** Department of Ophthalmology, Guangdong General Hospital, Guangdong Academy of Medical Sciences, No. 106, The second zhongshan road, Guangzhou, 510080 Guangdong China

**Keywords:** Cytomegalovirus, Glaucoma, Anterior uveitis, Posner–Schlossman syndrome (PSS), Immunocompetent

## Abstract

**Background:**

Cytomegalovirus can cause ocular anterior uveitis with ocular hypertension. Basis on the therapy, ocular pressure usually can be controlled. We report a case of a man who had unilateral cytomegalovirus anterior uveitis with refractory glaucoma during the process of treatment.

**Case presentation:**

A 57-year-old man who was diagnosed Posner–Schlossman syndrome and was admitted for repeatly attacks of raised IOP in left eye for 4 months. We found the cytomegalovirus -DNA was high (1800 copies/ml) in his aqueous. After systemic used of antiviral drug accompany with topical used of anti-inflammation, anti-glaucoma agents and genciclovir gel, the ocular pressure was dropped to normal. While the pressure elevated again in a month after stopping systemic antiviral treatment. Furthermore, the second test showed cytomegalovirus in aqueous humor decreased to 526 copies/ml. Intravenous drugs to antiviral, anti-inflammatory and anti-glaucoma were applied, but the ocular pressure was still high. In the progression of glaucomatous damage in the eye, glaucoma surgery was operated with no cytomegalovirus was detected. At last, the postoperative ocular pressure has been controlled.

**Conclusions:**

CMV infection is not rare. Patients have unilateral mild anterior inflammation with relapsed attacks of elevated intraocular pressure should be considered for CMV infection. We found that concurrent use of systemic and topical ganciclovir in a short period could reduce ocular CMV significantly, while ocular hypertension recurred. The antiviral treatment should be individualized. Glaucoma surgery could be offered to protect CEC loss and glaucomatous damage.

**Electronic supplementary material:**

The online version of this article (10.1186/s12886-018-0917-9) contains supplementary material, which is available to authorized users.

## Background

Cytomegalovirus (CMV), an opportunistic virus in the Herpesviridae family, is known as a main cause of ocular infection such as CMV retinitis in immunecompromised patients [1, 2]. However, recently studies have shown that CMV infection can cause hypertensive anterior uveitis (AU), which presents in acute or chronic inflammation types, in immunecompetent individuals due to the progress of PCR analysis of CMV-DNA from human aqueous samples [2–8]. For these patients, antiviral is essential but the treatment durations are various. The intraocular pressure (IOP) usually comes down after treatment. We report a case of AU with acute refractory glaucoma in a male patient with cytomegalovirus infection in unilateral eye.

## Case presentation

**Fig. 1:**
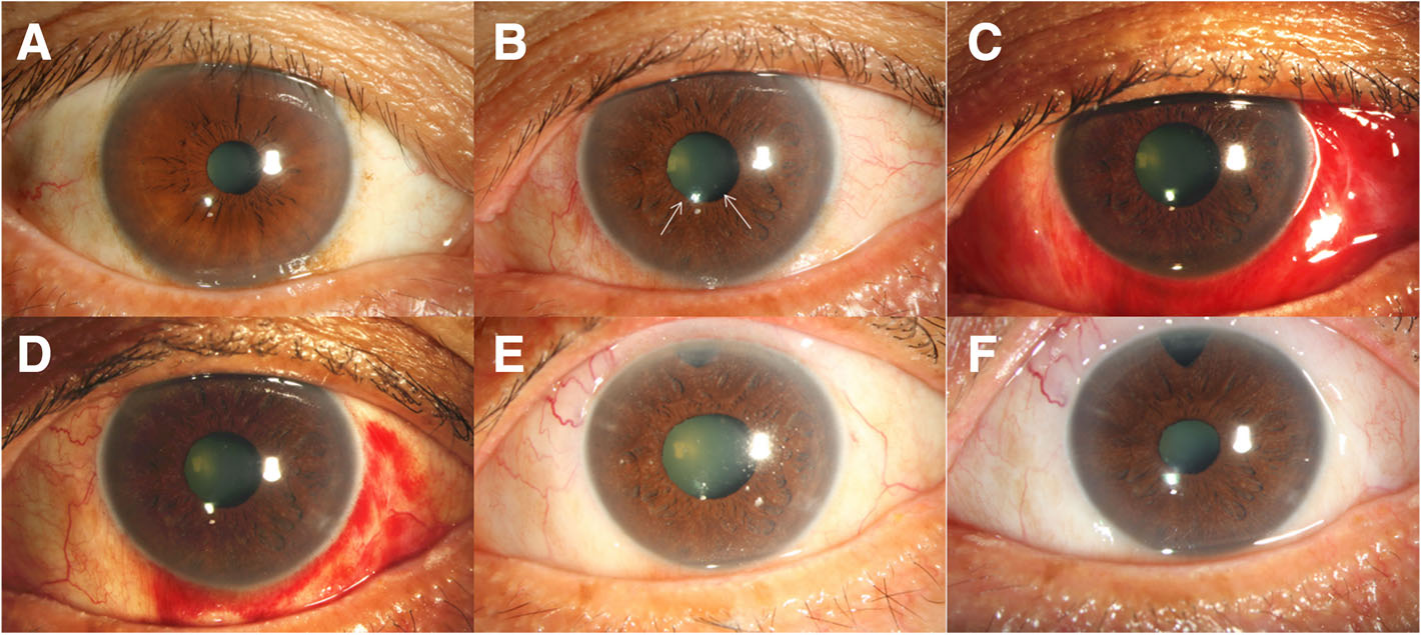
Photographs taken at different follow-up periods. **a** Clear cornea and iris of right eye before admission. **b** Clear cornea with a few small to medium Keratic precipitates (KPs) (white arrows), a mild anterior chamber inflammation and patched iris atrophy of left eye before admission. **c** Recurred ocular pressure with mild corneal edema and few small KPs of left eye during the first hospitalization. **d** Elevated ocular pressure with mild corneal edema in left eye during the second hospitalization. **e** Clear cornea of left eye after trabeculectomy. **f** The left eye 1 year postoperatively

**Fig. 2:**
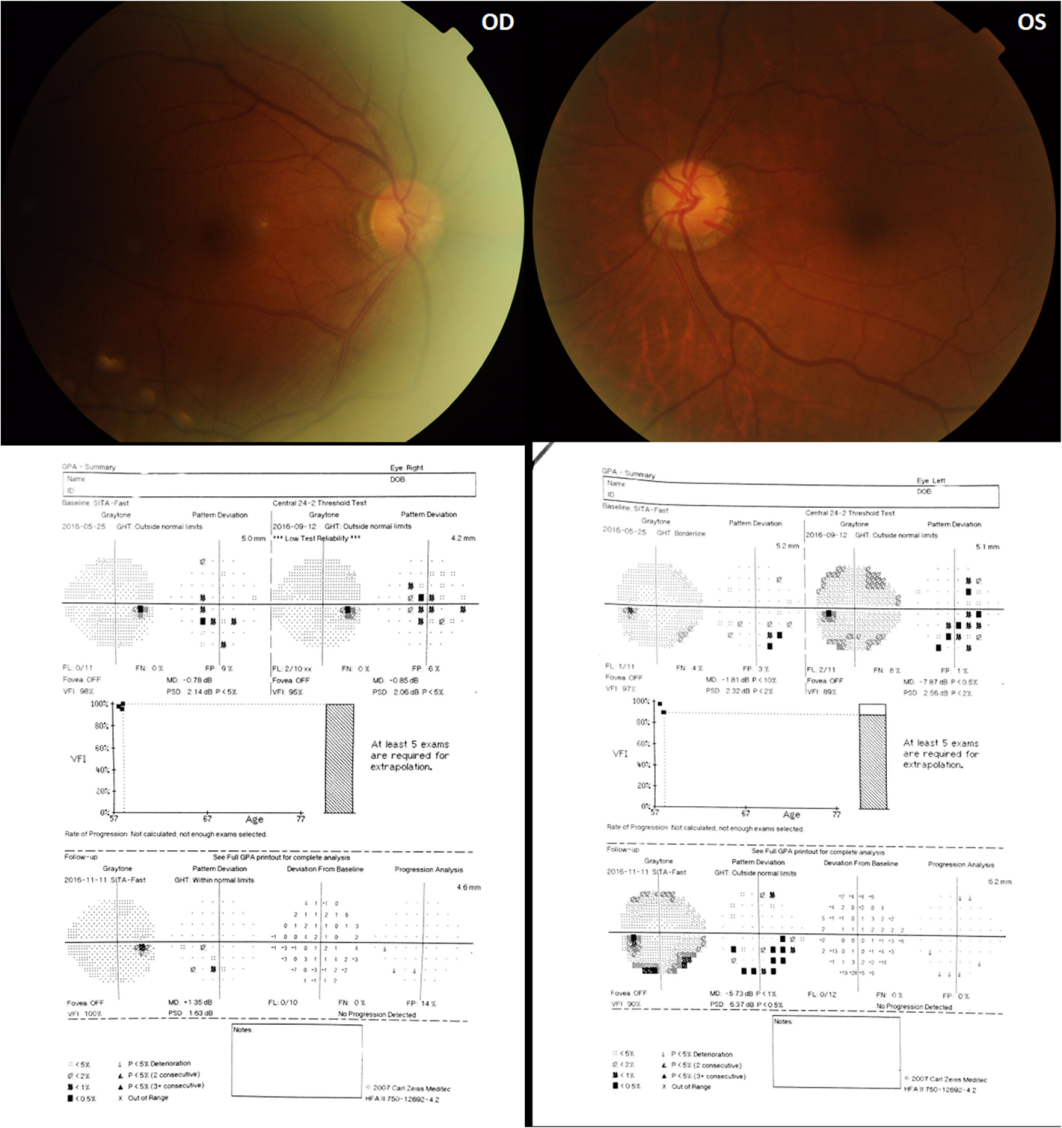
Bilateral fundal photographs and the change of visual fields at three different follow-up times (the first visit in outpatients center; during the first hospitalization; before trabeculectomy). OD: Normal optic disc and no obvious glaucomatous sight defect. OS: Enlarged optic disk area with visual field defects loss at inferior quadrant

**Fig. 3:**
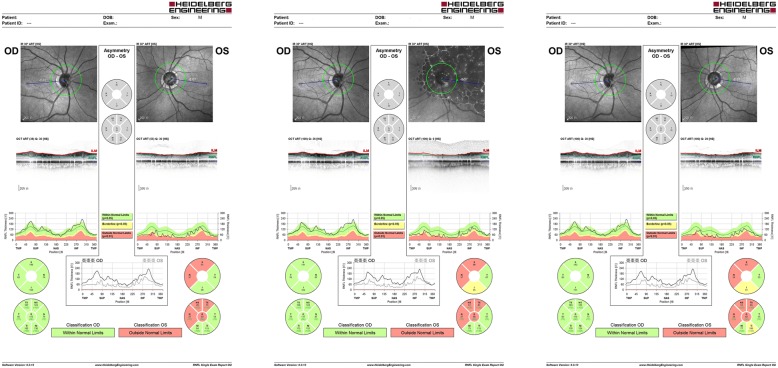
Bilateral retinal nerve fiber layer thickness examinations at three different follow-up times (the first visit in outpatients center; during the first hospitalization; before trabeculectomy). OD: All within normal limits. OS: Inferior quadrant becomes thinner over time

A 57-year-old man was admitted to our institution with repeated attacks of high IOP accompanied with AU in left eye for 4 months in 2016. He had a history of Posner–Schlossman syndrome (PSS) in left eye with recurred several times 20 years ago. According to outpatient medical records (Figs. 1a, b, 2 and 3a; Additional files 1, 2 and 3), the maximum IOP is 36 mmHg and the best-corrected visual acuity (BCVA) is 20/25 with deep anterior chamber, fine pupillary light reflex and a few anterior chamber inflammations during 4-month follow up period. Examinations found significant thinned retinal nerve fiber layer (RNFL) at superior and nasal side corresponding to visual field defects in inferior temple quadrant for the infected eye on his first visit in our outpatient center in May 2016. Topical corticosteroids and anti-glaucoma agents were treated then while IOP elevated repeatedly.

On admission in September, his BCVA is 20/25 for right eye, and 20/200 for left eye. The IOP is 18 mmHg and 40 mmHg respectively. The main features in his left eye are cornea edema with bullous keratopathy, fine white keratic precipitates (KPs), deep anterior chamber, 2+ flare counts in aqueous and patched iris atrophy. Meanwhile, visual field defects and glaucomatous optic nerve defects were enlarged (Figs. 2 and 3b). Quantitative PCR testing for CMV-DNA was immediately performed in samples from aqueous humor and serum. Human immunodeficiency virus (HIV) and other infection antibodies in serum were also tested. Results showed serum IgG for CMV but no IgM, HIV and other infectious diseases. The CMV-DNA in aqueous sample was positive (1800 copies/ml). Then the patient was treated with 2-week systemic antiviral therapy (ganciclovir 5 mg/kg twice a day intravenously for a week, followed by once a day for another week), topical 0.15% ganciclovir gel four times daily, 1% prednisolone acetate eye drops (Pred Forte) four times daily and local anti-glaucoma medications (brinzolamide and brimonidine). The IOP became normal 3 days later and the patient was asked to follow up.

**Fig. 4:**
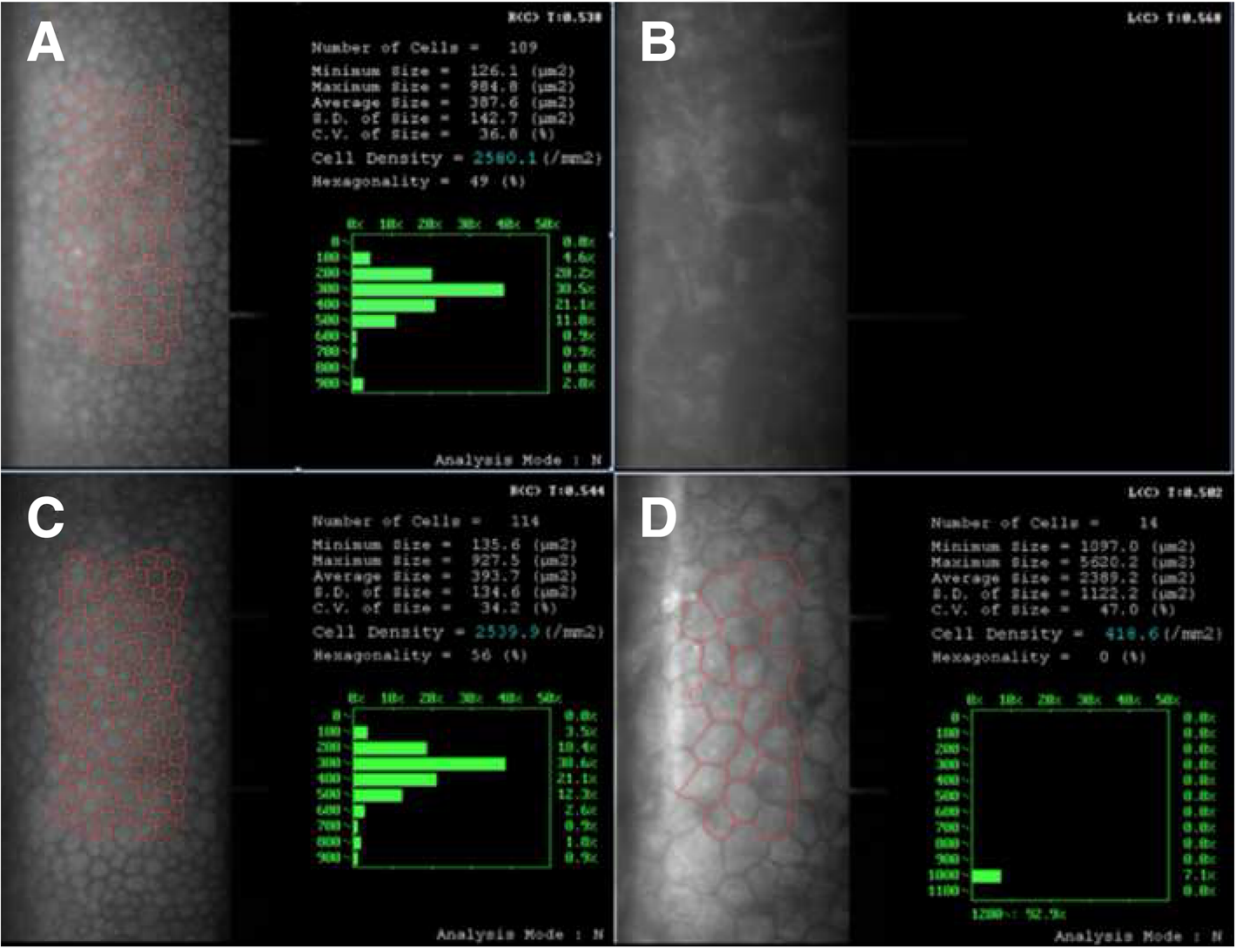
Bilateral endothelial cells count showed cornea endothelial cells density is lower in left eye. **a** The endothelial cells count of right eye before trabeculectomy. **b** The endothelial cells count of left eye before trabeculectomy. **c** The endothelial cells count of right eye 6 months postoperatively. **d** The endothelial cells count of left eye 6 months postoperatively

However, 12 days after cessation of systemic use of genciclovir, the IOP in patient’s left eye reached to 33 mmHg with topical genciclovir gel, corticosteroid (Pred Forte) and NSAID (Pranoprofen) consistently used (Fig. 1c). The patient was readmitted because of the persistence of elevated IOP despite additional medication treatments for 20 days. After readmission, test showed that CMV-DNA was only 526 copies/ml in aqueous. But the IOP maintained a high level of 50 mmHg or above (Fig. 1d). To exclude corticosteroid glaucoma, NSAID were applied topically without steroid, accompanied with genciclovir gel and glaucoma medications. In addition, systemic therapies were administered for anti-virus (ganciclovir 5 mg/kg twice a day intravenously), anti-inflammatory (vein injection of methylprednisolone 80 mg per day) and anti-glaucoma (anterior chamber penetration, intravenous mannitol) in subsequent 15-day. Nevertheless, in view of the persistence of high IOP, corneal opacity with no cornea endothelial cells (CEC) could be measured and the development of glaucomatous damage (Figs. 2, 3, and 4a, b), trabeculectomy was operated for refractory glaucoma (Fig. 1e) of left eye in Nov. 2016. At this time, the test for CMV-DNA of the eye was negative (10 copies/ml) and the postoperative IOP has been controlled (8-13 mmHg) with BCVA of 20/25 a year later (Fig. 1f). However, the count of CEC in the infected eye has much lesser than that in the fellow eye after 6 months (Fig. 4c, d; Additional file 4).

## Discussion

To data, CMV can cause an acute, recurrent, or chronic hypertensive anterior uveitis, particularly in the middle aged Asian male populations [3, 5, 6]. In acute CMV-AU, it often been clinical diagnosed as PSS or glaucomatocy clitic crisis, which typically characterized by recurrent pathological ocular hypertension with mild anterior segment inflammation [5–9]. And in chronic course, the manifestations were insidious persistent, with slightly elevated IOP, lower aqueous flare counts and mild ocular blurring [2, 9]. Though there were no definite clinical manifestations that enabled us to distinguish the eyes with CMV infected or not, there are some characteristic features indicated CMV infection: coin-shaped KPs, nodular endothelial lesions and patched or diffused iris atrophy [3, 5, 9–13]. Meanwhile, Posner-Schlossman syndrome (PSS) was described as acute attacks of unilateral, nongranulomatous, mild anterior uveitis that accompanied by markedly elevated IOP and opened anterior chamber [14]. Usually, it always a benign disease and rarely causes intractable glaucoma and glaucomatous optic neuropathy [15]. Thus, it is important to note that any eye with hypertensive anterior uveitis exhibits endotheliitis, KPs or iris atrophy, especially with optic nerve damage, qualitative PCR analysis of aqueous for viruses DNA should be performed as the presumptive evidence of viral infection.

CMV-AU treatment should be customized due to the severity of the disease. The common management focused on anti-virus and control elevated IOP [3, 5, 6]. Topical use of ganciclovir gel is well responded with minimal side effect and economized in CMV infections. But the ocular hypertension with inflammation often recurred frequently. Various modalities of ganciclovir (or its prodrug valganciclovir) including oral application and intravitreal implantation/injection were used [5–9]. Most studies recommend a 3-month course of oral anti-viral treatment [3, 5, 6, 16]. Nevertheless, some studies reported that the relapse rate in patients with CMV were high regardless of treatment regimen [6, 17]. It was anticipated that antiviral medicines like ganciclovir are virostatic. It could reduce viral replication but could not eradicate it [3, 6, 16, 18]. For this reason, a prolonged course of systemic antivirals was reported [3, 16, 18].

The virus load in aqueous humor maybe one of the reasons for the relapse of CMV induced hypertensive AU. In our case, after 14 days systemic using of ganciclovir combined with topical ganciclovir gel four times a day consistently, the CMV DNA copies from the aqueous humor was much lower than that in the first time when uncontrolled IOP relapsed with little aqueous flare. Some studies presumed that the impairment of trabecular meshwork function was involved [7–9, 16, 19]. A study proved that human TM cells can support CMV replication in vitro effectively and the active CMV viral infection in TM cells which may be the key mechanism for the elevation of IOP in anterior viral uveitis [20]. Other than that, ocular immune response may also play a role in the relapse of ocular hypertension [19]. The duration of systemic and topical antiviral therapy should be reconsidered. Further studies are needed to investigate detail mechanisms.

Glaucoma surgery is preferable for patients with consecutive uncontrolled IOP. Pathological IOP elevation is the main reason for vision loss in CMV infection cases [6–8]. Studies show that CMV associated AU is particularly at risk for the frequent attacks of high IOP [7–9, 18], which leads to a series of secondary glaucomatous damages. Hence, treatment may be initiated with anti-glaucoma agents as a supplement. But considering the toxicity of long-term use of topical medicines, advanced surgical treatment, such as trabeculectomy, is required to protect progressive visual field defects in patients with refractory ocular hypertension [4–9, 16, 18]. On the other hand, consistent with our study, a number of studies have shown that CEC loss appears to be significantly correlated with CMV viral load [4, 10, 12]. Meanwhile, repeated relapse of IOP leads to CEC decrease [7]. Glaucoma surgery could irrigate CMV in aqueous humor and stabilize IOP, which prevents CEC dysfunction and glaucomatous optic nerve damage.

## Conclusions

CMV infection is not rare. Patients have unilateral mild anterior inflammation with relapsed attacks of pathological elevated IOP should be considered for CMV infection. We report a CMV infected case with repeatly elevated IOP and found out that concurrent use of systemic and topical ganciclovir in a short duration could reduce ocular CMV effectively. In view of the IOP increased with spikes may not correlate with viral load in aqueous humor, the antiviral treatment duration should be individualized. Glaucoma surgery could be offered to protect CEC loss and glaucomatous damage when ocular hypertension attacks frequently with progressive visual loss.

## Additional files

Additional file 1: Raw data-figure1-A: Picture of right eye before admission. Raw data-figure1-B: Picture of left eye before admission. Raw data-figure1-C: Picture of left eye during the first hospitalization. Raw data-figure1-D: Picture of left eye during the second hospitalization. Raw data-figure1-E: Picture of left eye after trabeculectomy. Raw data-figure1-F: Picture of left eye 1 year postoperatively. (ZIP 2973 kb)

Additional file 2: Raw data-figure2-A: Fundus photograph of right eye. Raw data-figure2-B: Fundus photograph of left eye. Raw data-figure2-C: Visual fields results of right eye at three different follow-up times (the first visit in outpatients center; during the first hospitalization; before trabeculectomy). Raw data-figure2-D: Visual fields results of left eye at three different follow-up times (the first visit in outpatients center; during the first hospitalization; before trabeculectomy). (ZIP 12863 kb)

Additional file 3: Raw data-figure3-A: Bilateral retinal nerve fiber layer thickness examinations at the first visit in outpatients center. Raw data-figure3-B: Bilateral retinal nerve fiber layer thickness examinations during the first hospitalization. Raw data-figure3-C: Bilateral retinal nerve fiber layer thickness examinations before trabeculectomy. (ZIP 4091 kb)

Additional file 4: Raw data-figure4-A: The report of endothelial cells count of right eye before trabeculectomy. Raw data-figure4-B: The report of endothelial cells count of left eye before trabeculectomy. Raw data-figure4-C: The report of endothelial cells count of right eye 6 months postoperatively. Raw data-figure4-D: The report of endothelial cells count of left eye 6 months postoperatively. (ZIP 480 kb)

## Abbreviations

AU: Anterior uveitis
BCVA: Best-corrected visual acuity
CEC: Cornea endothelial cells
CMV: Cytomegalovirus
HIV: Human immunodeficiency virus
IOP: Intraocular pressure
KPs: Keratic precipitates
RNFL: Retinal nerve fiber layer
