# Supplementary figures and images for: Cytomegalovirus-related uncontrolled glaucoma in an immunocompetent patient: a case report and systematic review
Source: BMC Ophthalmol. 2018 Sep 29;18:259. doi: 10.1186/s12886-018-0917-9 (PMC6162942; doi:10.1186/s12886-018-0917-9)

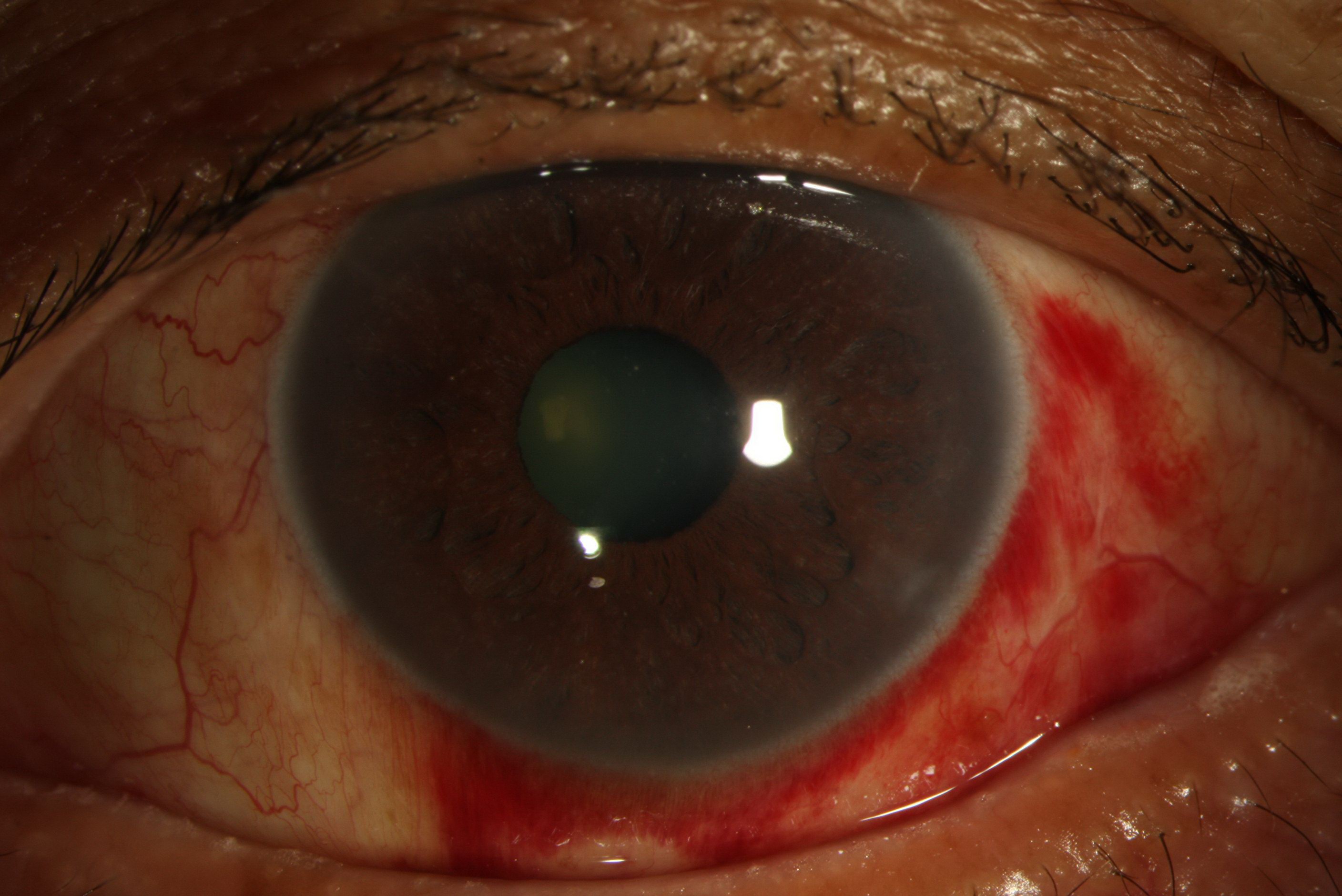

Supplement: Supplementary file 1 — Raw data-figure1-A: Picture of right eye before admission. Raw data-figure1-B: Picture of left eye before admission. Raw data-figure1-C: Picture of left eye during the first hospitalization. Raw data-figure1-D: Picture of left eye during the second hospitalization. Raw data-figure1-E: Picture of left eye after trabeculectomy. Raw data-figure1-F: Picture of left eye 1 year postoperatively. (ZIP 2973 kb) [file 12886_2018_917_MOESM1_ESM.zip › D(11-11-os)R2.jpg]

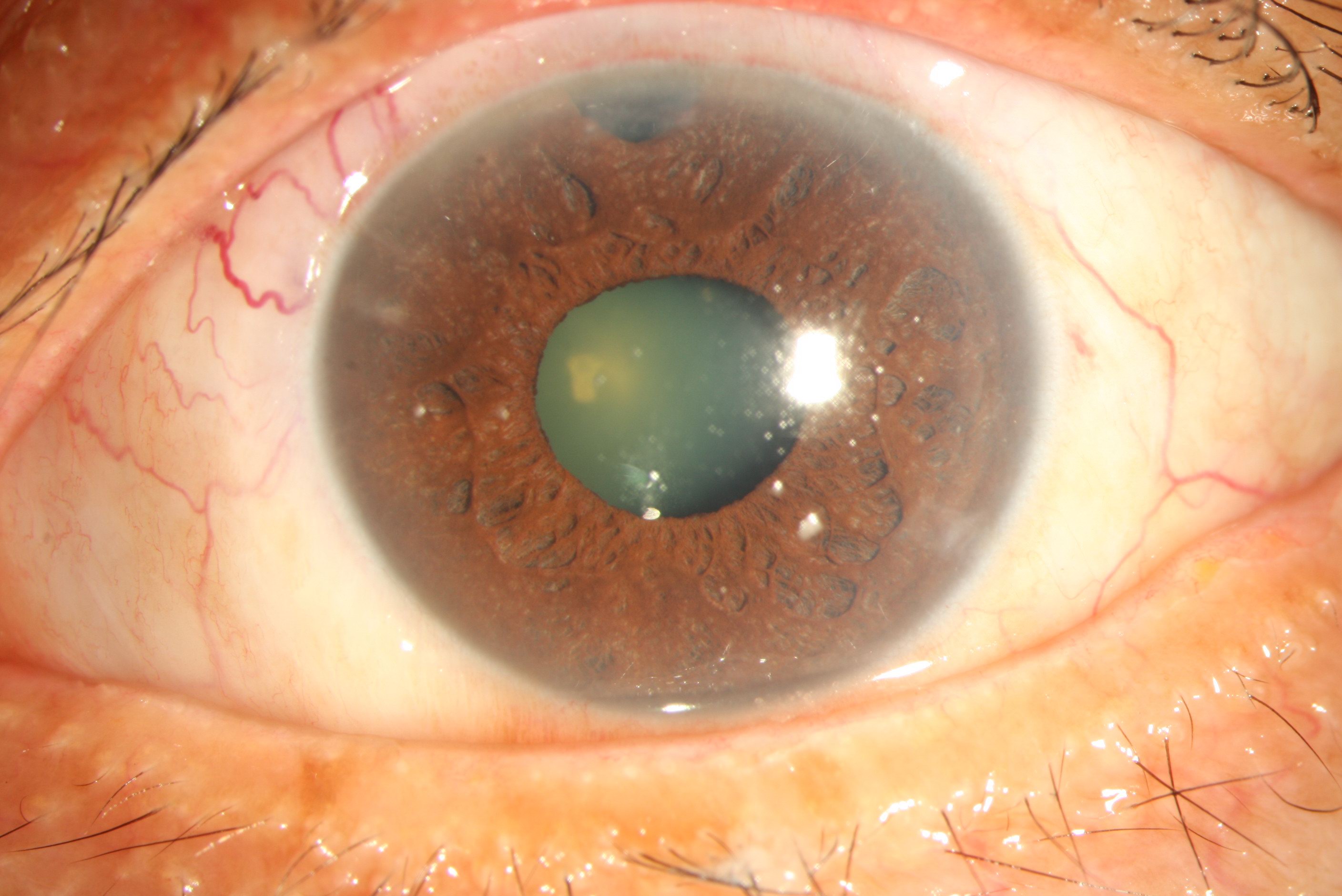

Supplement: Supplementary file 1 — Raw data-figure1-A: Picture of right eye before admission. Raw data-figure1-B: Picture of left eye before admission. Raw data-figure1-C: Picture of left eye during the first hospitalization. Raw data-figure1-D: Picture of left eye during the second hospitalization. Raw data-figure1-E: Picture of left eye after trabeculectomy. Raw data-figure1-F: Picture of left eye 1 year postoperatively. (ZIP 2973 kb) [file 12886_2018_917_MOESM1_ESM.zip › E(12-19-os)R2.jpg]

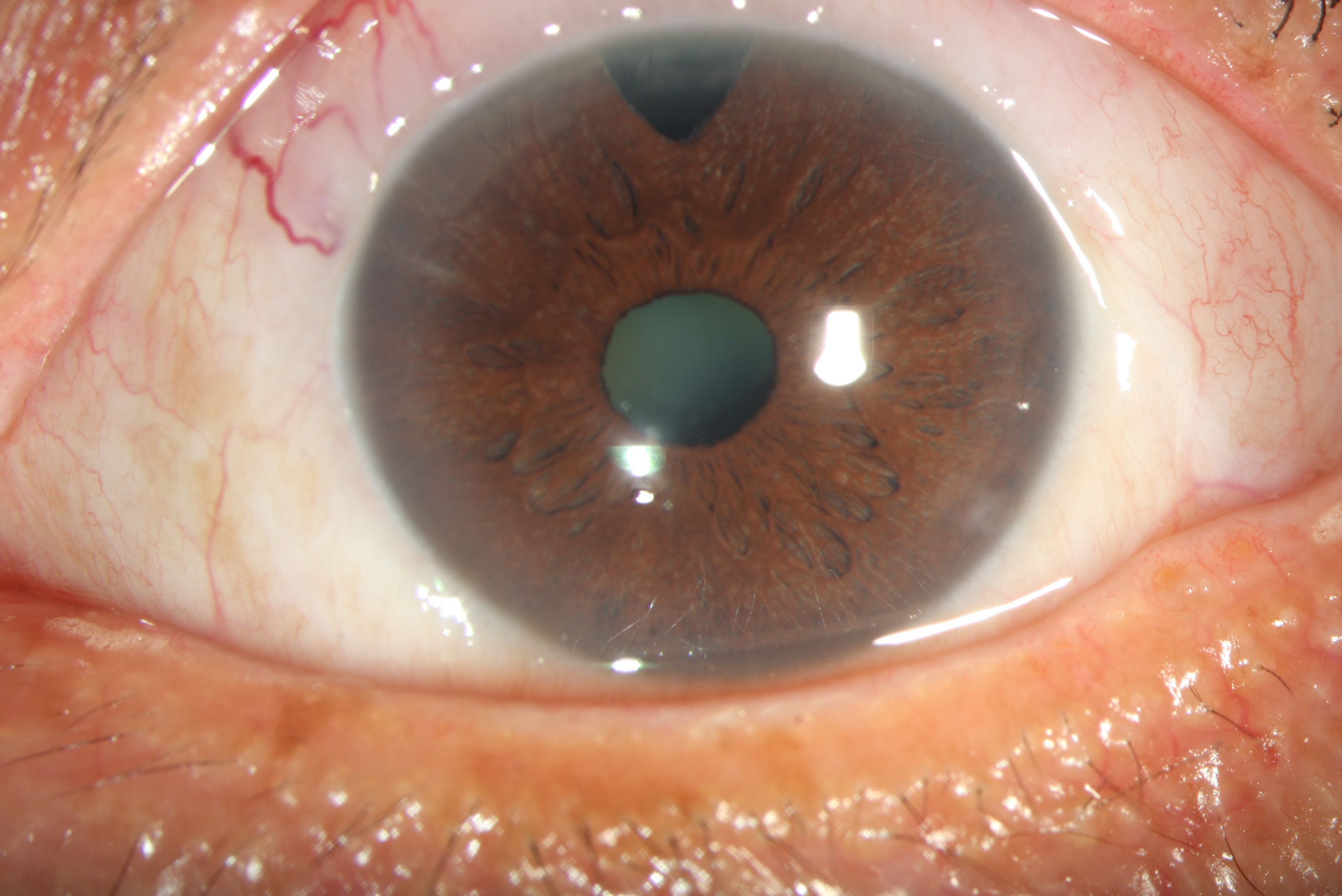

Supplement: Supplementary file 1 — Raw data-figure1-A: Picture of right eye before admission. Raw data-figure1-B: Picture of left eye before admission. Raw data-figure1-C: Picture of left eye during the first hospitalization. Raw data-figure1-D: Picture of left eye during the second hospitalization. Raw data-figure1-E: Picture of left eye after trabeculectomy. Raw data-figure1-F: Picture of left eye 1 year postoperatively. (ZIP 2973 kb) [file 12886_2018_917_MOESM1_ESM.zip › F(5-4-os)R2.jpg]

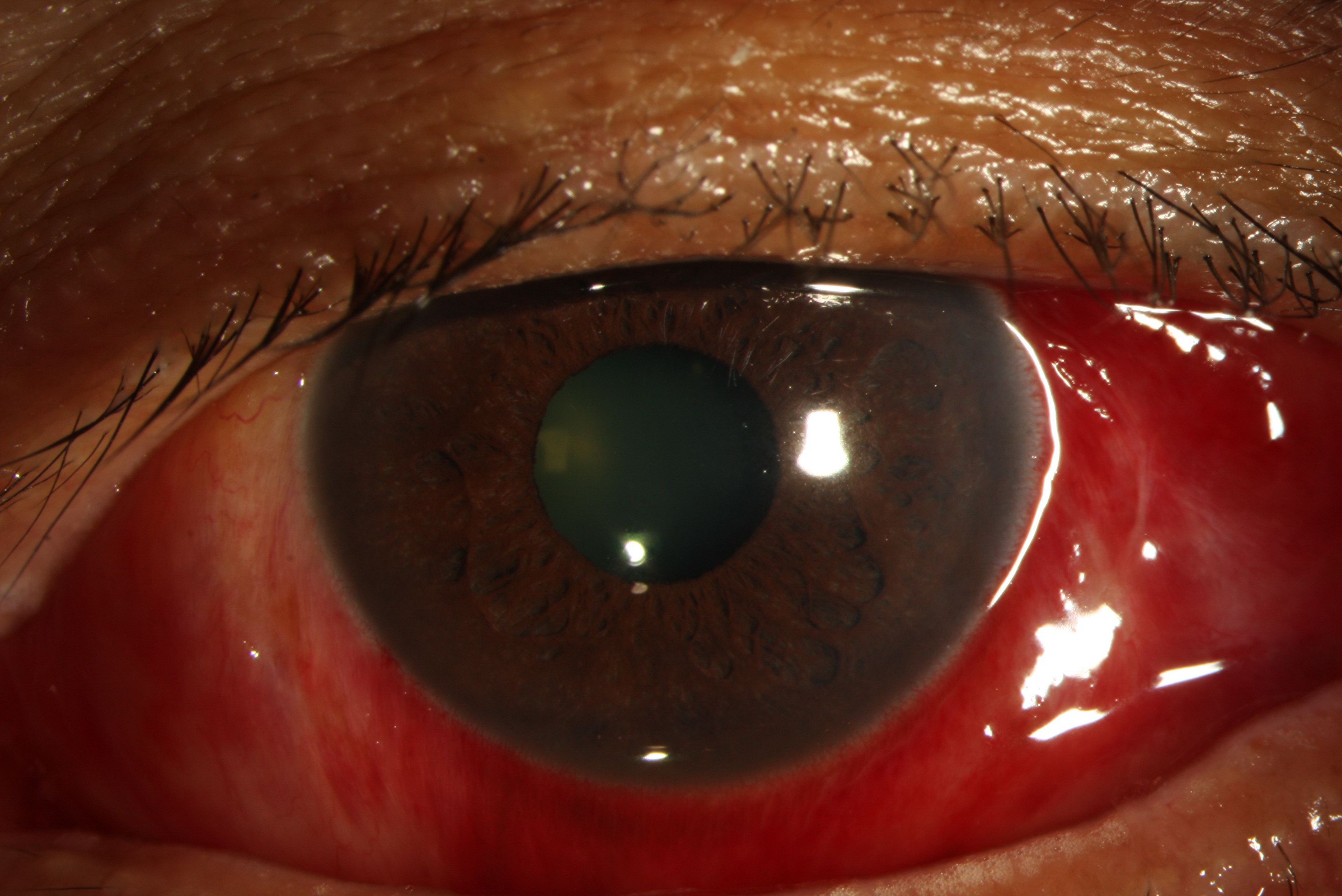

Supplement: Supplementary file 1 — Raw data-figure1-A: Picture of right eye before admission. Raw data-figure1-B: Picture of left eye before admission. Raw data-figure1-C: Picture of left eye during the first hospitalization. Raw data-figure1-D: Picture of left eye during the second hospitalization. Raw data-figure1-E: Picture of left eye after trabeculectomy. Raw data-figure1-F: Picture of left eye 1 year postoperatively. (ZIP 2973 kb) [file 12886_2018_917_MOESM1_ESM.zip › renamed_3ad3fR2.jpg]

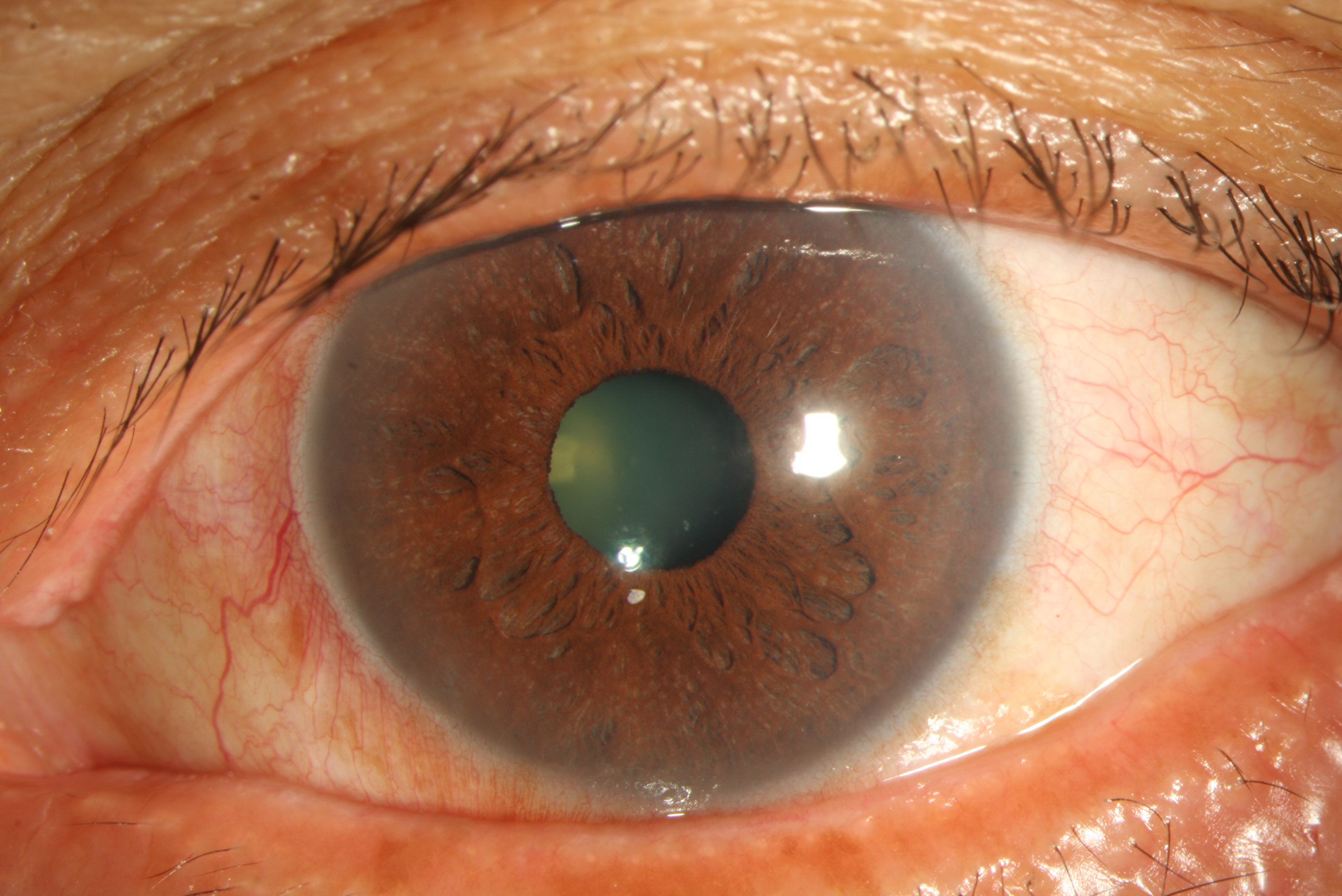

Supplement: Supplementary file 1 — Raw data-figure1-A: Picture of right eye before admission. Raw data-figure1-B: Picture of left eye before admission. Raw data-figure1-C: Picture of left eye during the first hospitalization. Raw data-figure1-D: Picture of left eye during the second hospitalization. Raw data-figure1-E: Picture of left eye after trabeculectomy. Raw data-figure1-F: Picture of left eye 1 year postoperatively. (ZIP 2973 kb) [file 12886_2018_917_MOESM1_ESM.zip › renamed_ba5dcR2.jpg]

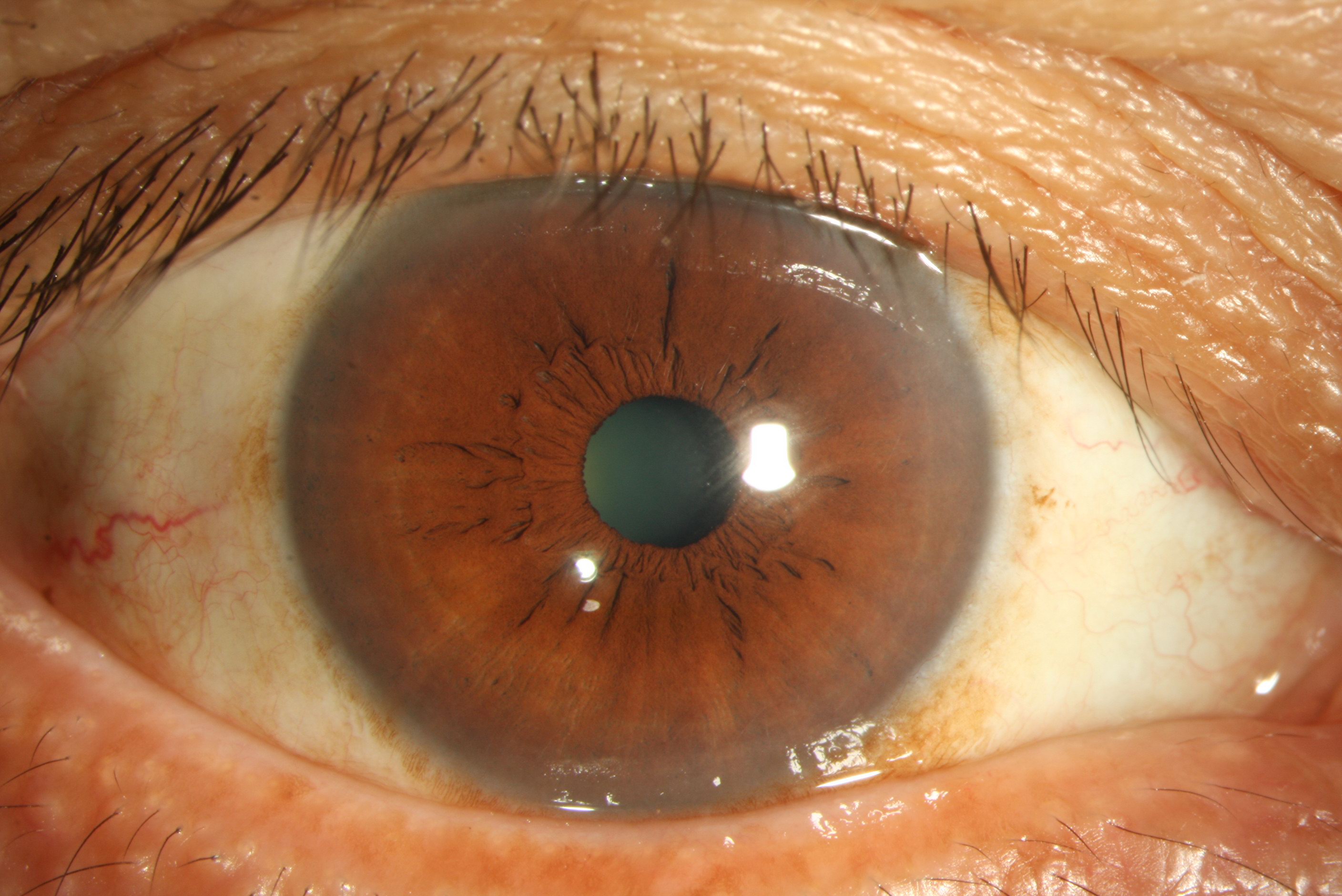

Supplement: Supplementary file 1 — Raw data-figure1-A: Picture of right eye before admission. Raw data-figure1-B: Picture of left eye before admission. Raw data-figure1-C: Picture of left eye during the first hospitalization. Raw data-figure1-D: Picture of left eye during the second hospitalization. Raw data-figure1-E: Picture of left eye after trabeculectomy. Raw data-figure1-F: Picture of left eye 1 year postoperatively. (ZIP 2973 kb) [file 12886_2018_917_MOESM1_ESM.zip › renamed_fea5dR2.jpg]

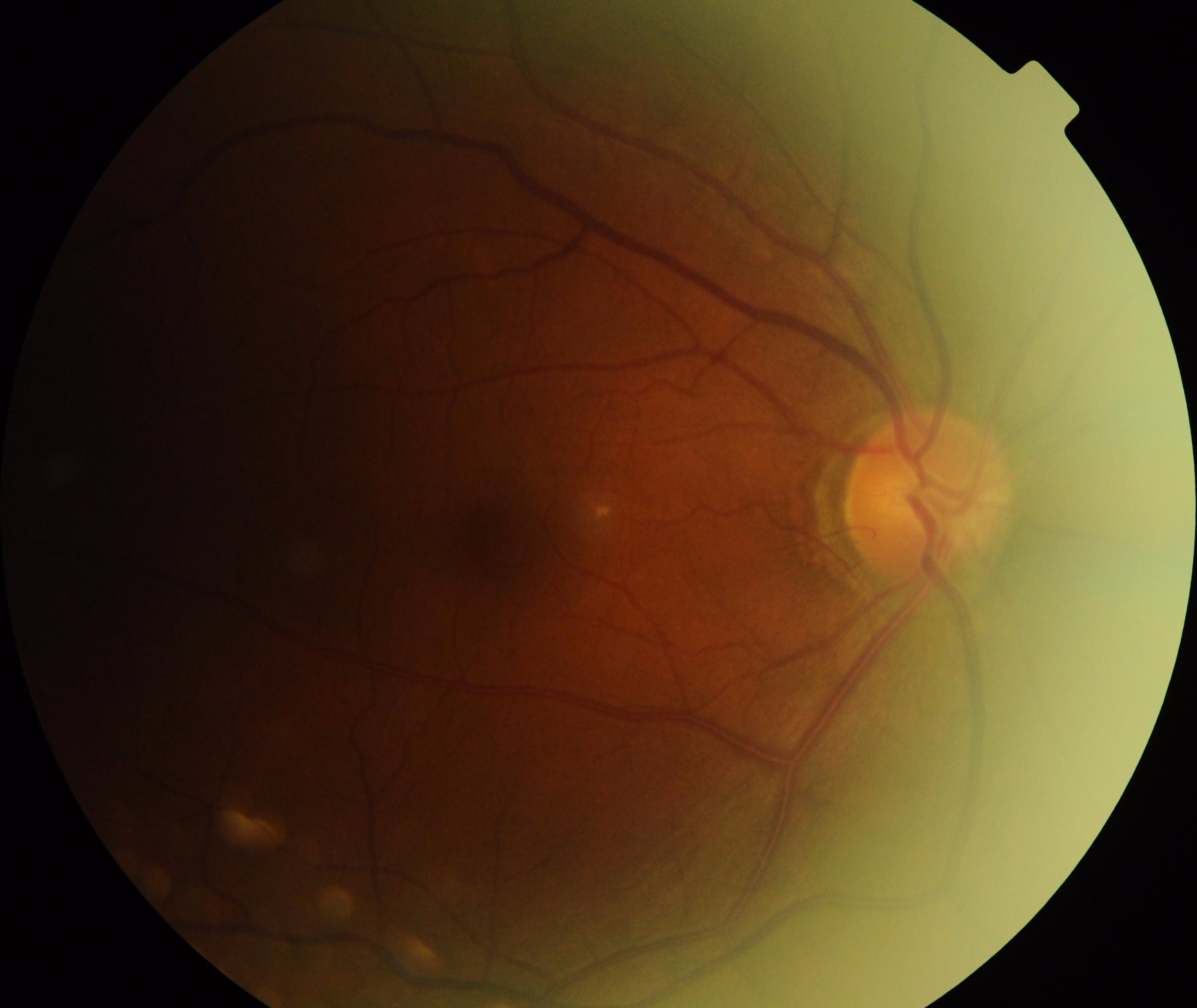

Supplement: Supplementary file 2 — Raw data-figure2-A: Fundus photograph of right eye. Raw data-figure2-B: Fundus photograph of left eye. Raw data-figure2-C: Visual fields results of right eye at three different follow-up times (the first visit in outpatients center; during the first hospitalization; before trabeculectomy). Raw data-figure2-D: Visual fields results of left eye at three different follow-up times (the first visit in outpatients center; during the first hospitalization; before trabeculectomy). (ZIP 12863 kb) [file 12886_2018_917_MOESM2_ESM.zip › 2016-11-1-odR2.tif]

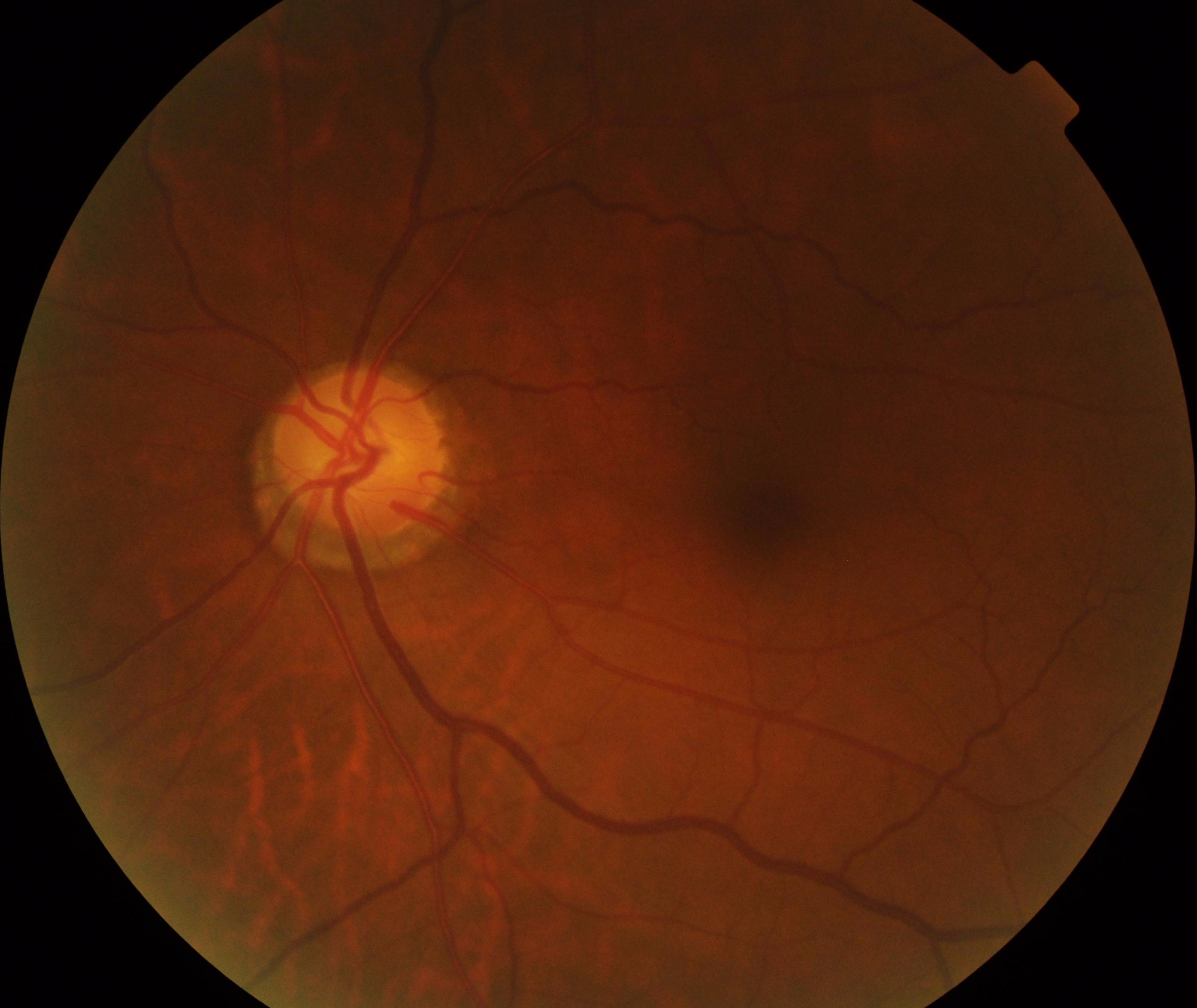

Supplement: Supplementary file 2 — Raw data-figure2-A: Fundus photograph of right eye. Raw data-figure2-B: Fundus photograph of left eye. Raw data-figure2-C: Visual fields results of right eye at three different follow-up times (the first visit in outpatients center; during the first hospitalization; before trabeculectomy). Raw data-figure2-D: Visual fields results of left eye at three different follow-up times (the first visit in outpatients center; during the first hospitalization; before trabeculectomy). (ZIP 12863 kb) [file 12886_2018_917_MOESM2_ESM.zip › 2016-11-1-osR2.tif]

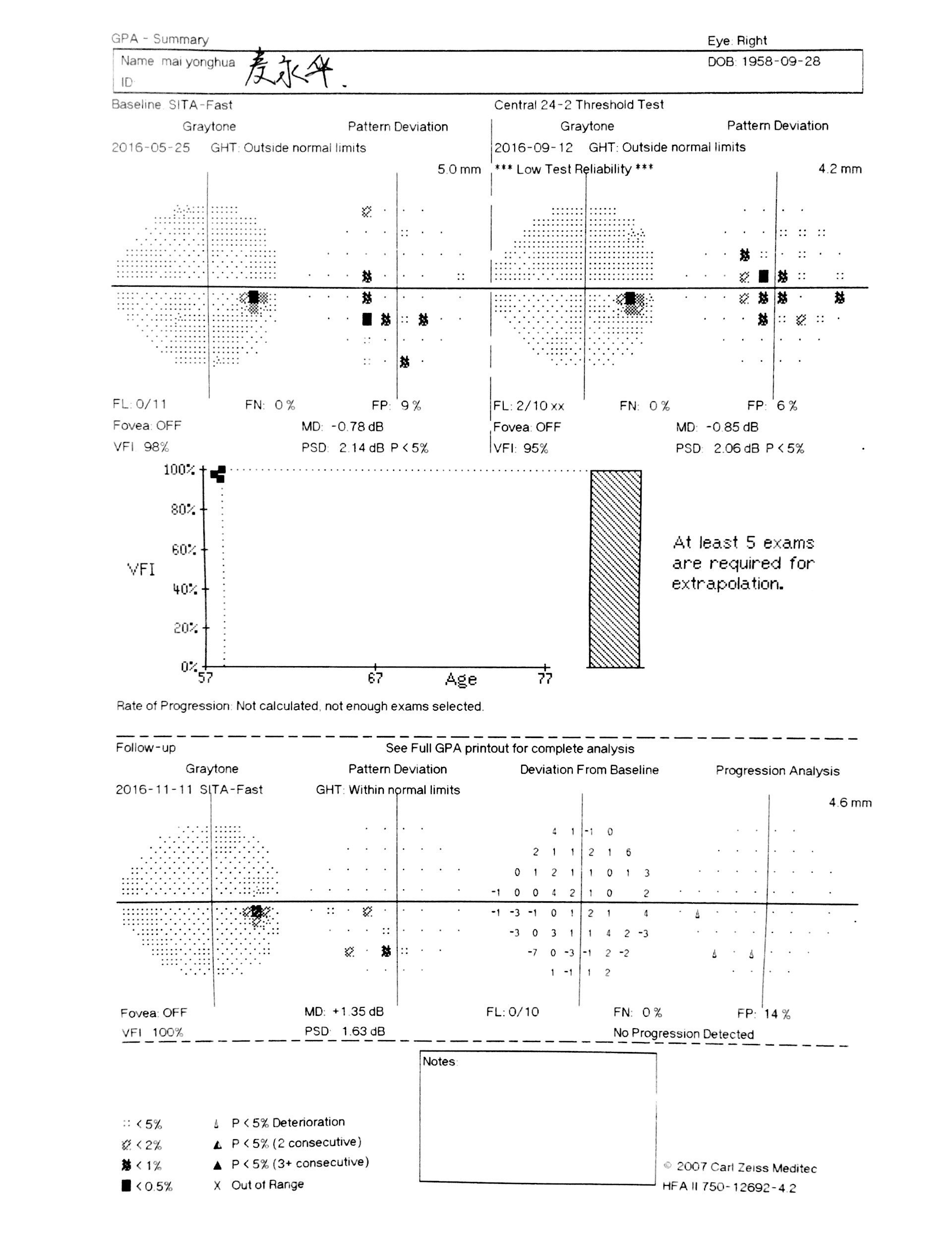

Supplement: Supplementary file 2 — Raw data-figure2-A: Fundus photograph of right eye. Raw data-figure2-B: Fundus photograph of left eye. Raw data-figure2-C: Visual fields results of right eye at three different follow-up times (the first visit in outpatients center; during the first hospitalization; before trabeculectomy). Raw data-figure2-D: Visual fields results of left eye at three different follow-up times (the first visit in outpatients center; during the first hospitalization; before trabeculectomy). (ZIP 12863 kb) [file 12886_2018_917_MOESM2_ESM.zip › odR2.png]

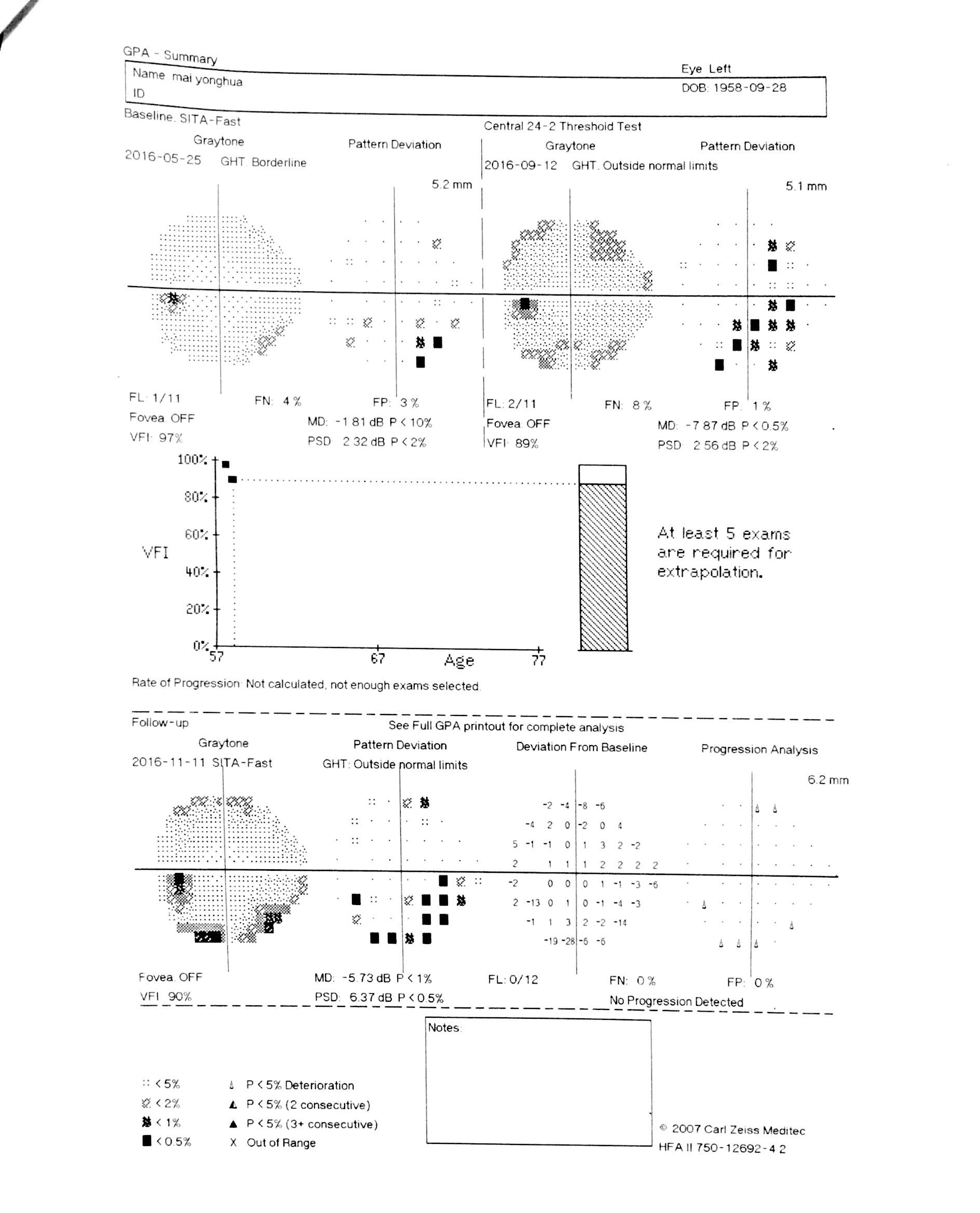

Supplement: Supplementary file 2 — Raw data-figure2-A: Fundus photograph of right eye. Raw data-figure2-B: Fundus photograph of left eye. Raw data-figure2-C: Visual fields results of right eye at three different follow-up times (the first visit in outpatients center; during the first hospitalization; before trabeculectomy). Raw data-figure2-D: Visual fields results of left eye at three different follow-up times (the first visit in outpatients center; during the first hospitalization; before trabeculectomy). (ZIP 12863 kb) [file 12886_2018_917_MOESM2_ESM.zip › osR2.png]

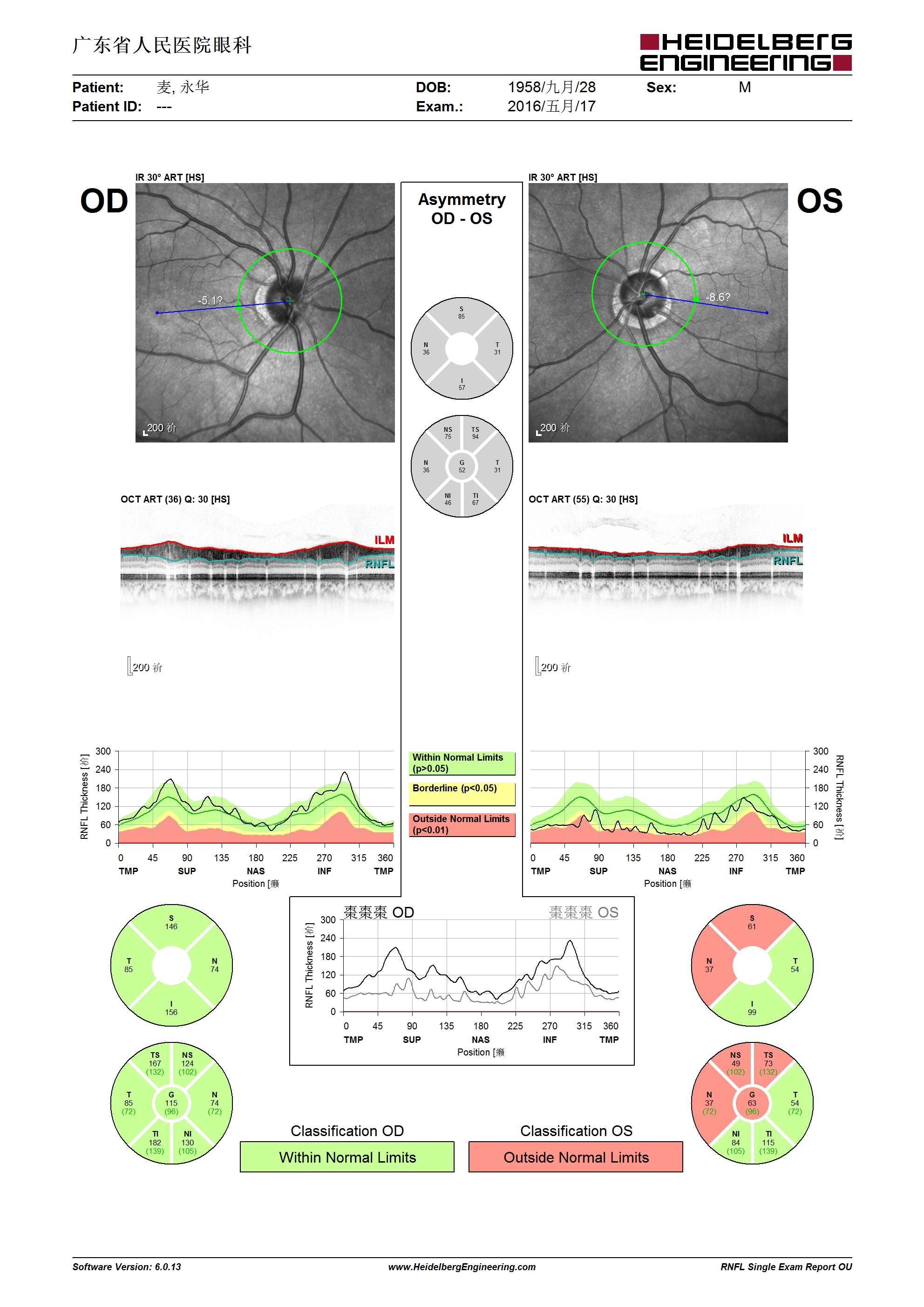

Supplement: Supplementary file 3 — Raw data-figure3-A: Bilateral retinal nerve fiber layer thickness examinations at the first visit in outpatients center. Raw data-figure3-B: Bilateral retinal nerve fiber layer thickness examinations during the first hospitalization. Raw data-figure3-C: Bilateral retinal nerve fiber layer thickness examinations before trabeculectomy. (ZIP 4091 kb) [file 12886_2018_917_MOESM3_ESM.zip › A.JPG]

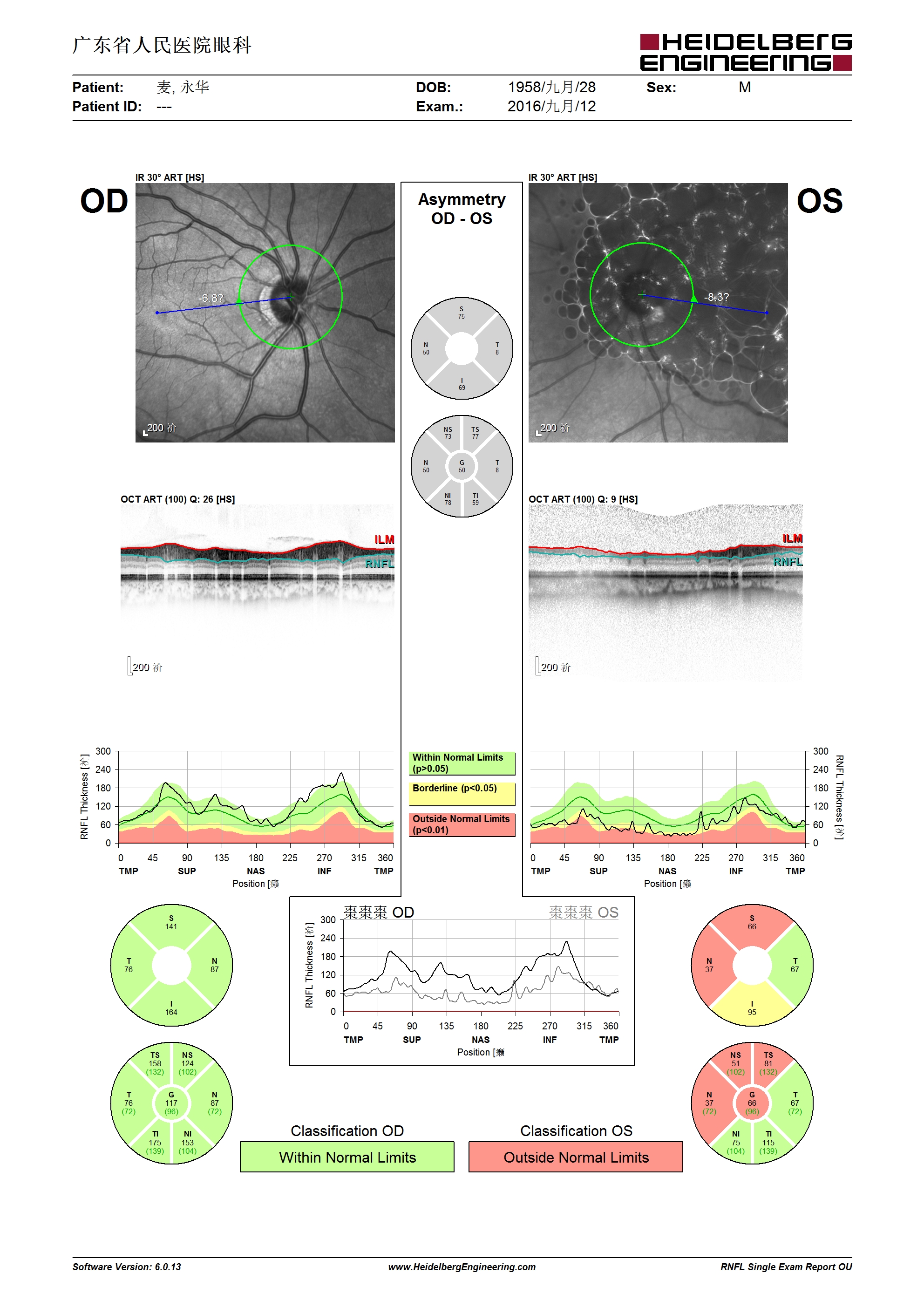

Supplement: Supplementary file 3 — Raw data-figure3-A: Bilateral retinal nerve fiber layer thickness examinations at the first visit in outpatients center. Raw data-figure3-B: Bilateral retinal nerve fiber layer thickness examinations during the first hospitalization. Raw data-figure3-C: Bilateral retinal nerve fiber layer thickness examinations before trabeculectomy. (ZIP 4091 kb) [file 12886_2018_917_MOESM3_ESM.zip › B.JPG]

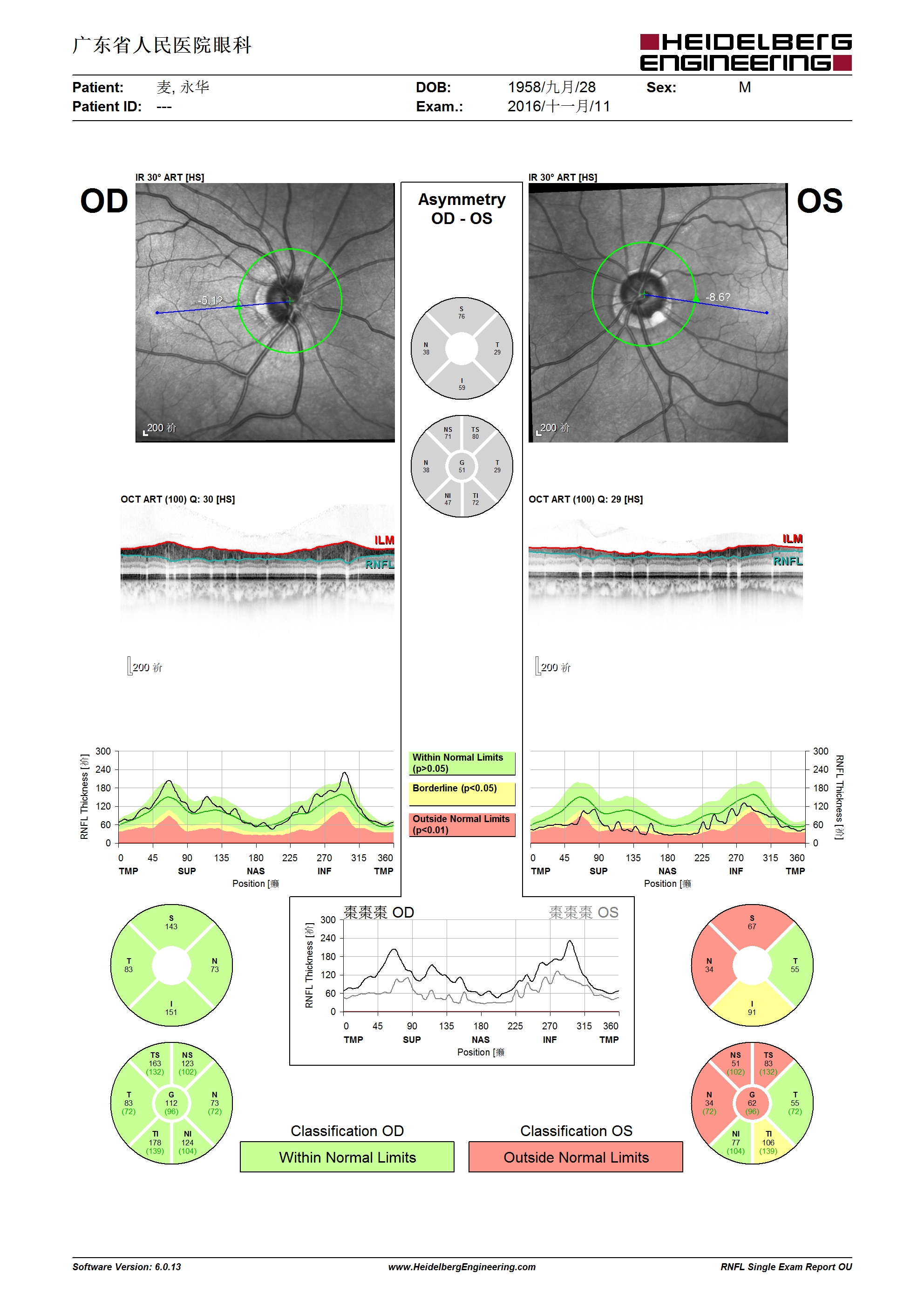

Supplement: Supplementary file 3 — Raw data-figure3-A: Bilateral retinal nerve fiber layer thickness examinations at the first visit in outpatients center. Raw data-figure3-B: Bilateral retinal nerve fiber layer thickness examinations during the first hospitalization. Raw data-figure3-C: Bilateral retinal nerve fiber layer thickness examinations before trabeculectomy. (ZIP 4091 kb) [file 12886_2018_917_MOESM3_ESM.zip › C.JPG]
