# Supplementary material for: Cytomegalovirus-related uncontrolled glaucoma in an immunocompetent patient: a case report and systematic review
Source: BMC Ophthalmol. 2018 Sep 29;18:259. doi: 10.1186/s12886-018-0917-9 (PMC6162942; doi:10.1186/s12886-018-0917-9)
Supplement: Supplementary file 4 — Raw data-figure4-A: The report of endothelial cells count of right eye before trabeculectomy. Raw data-figure4-B: The report of endothelial cells count of left eye before trabeculectomy. Raw data-figure4-C: The report of endothelial cells count of right eye 6 months postoperatively. Raw data-figure4-D: The report of endothelial cells count of left eye 6 months postoperatively. (ZIP 480 kb) [file 12886_2018_917_MOESM4_ESM.zip › renamed_5bc11R2.doc]

**广 东 省 人 民 医 院**

**角 膜 内 皮 细 胞 计 报 告 单**

| **姓 名** | 麦, 永华 | **编 号** | 012647 | **住院编号** |  |
| --- | --- | --- | --- | --- | --- |
| **性 别** | Male | **视 力** | VOD： VOS： | **检查日期** | 2016-09-02 |
| **年 龄** |  | **诊 断** | 虹膜睫状体炎 | **联系电话** |  |

**报告日期：2017-09-11**
